# Posterior Plating Through a Single Posterolateral Approach for a Comminuted Thurston-Holland Fragment in a Pediatric Triplane Ankle Fracture

**DOI:** 10.7759/cureus.116235

**Published:** 2026-09-14

**Authors:** Andrés Felipe Muñoz Leiva, Daniel Orlando Ibarra Alva

**Affiliations:** 1 Orthopedics and Traumatology, Hospital Juarez de México, Ciudad de México, MEX; 2 Orthopedics and Traumatology, Hospital Polanco Tecámac, Ciudad de México, MEX

**Keywords:** ankle fracture management, foot and ankle trauma, pediatric trauma, posterolateral approach, thurston-holland fragment, tillaux fracture, triplane fracture

## Abstract

We present the case of a 12-year-old boy who sustained a three-part triplane fracture of the left distal tibia following a torsional fall, with a small, comminuted posterior Thurston-Holland fragment. Triplane fractures are transitional injuries unique to mid-adolescence. While displaced fractures are conventionally managed through an anterior approach with indirect reduction of the posterior fragment, this technique relies on the posterior fragment being a single, congruent piece. In the present case, comminution of the posterior fragment precluded reliable indirect reduction, prompting a posterolateral surgical approach to achieve anatomical reduction under direct visualization.

The patient presented with pain, edema, deformity, and inability to ambulate, with the overlying skin tense and blanched over the deformity. Computed tomography confirmed a three-part triplane fracture comprising an anterolateral (Tillaux) fragment, a medial epiphyseal fragment, and a comminuted posterior metaphyseal fragment. The patient underwent open reduction and internal fixation performed entirely through a single posterolateral approach, with Kirschner-wire fixation of the posterior and Tillaux fragments and percutaneous screw fixation of the medial fragment. At final follow-up, the patient achieved an American Orthopaedic Foot and Ankle Society (AOFAS) Ankle-Hindfoot score of 99 out of 100 points, reflecting an excellent functional outcome. This case supports extrapolating posterior fixation principles well established in adult trimalleolar ankle fracture surgery to the pediatric transitional ankle fracture population and suggests that fragment morphology, rather than fracture classification alone, should guide the choice of surgical approach.

## Introduction

Triplane fractures are transitional injuries of the distal tibia that occur exclusively during mid-adolescence, coinciding with the asymmetric closure of the distal tibial physis, which proceeds centrally, then medially, and finally anterolaterally over approximately 18 months. These fractures account for approximately 5% to 15% of pediatric ankle fractures and are characterized by fracture lines propagating through three distinct planes - axial, sagittal, and coronal - typically resulting from a supination-external rotation or torsional mechanism [1,2].

Triplane fractures are classified according to the number of fracture fragments, most commonly as two-part, three-part, or four-part patterns. In the two-part variant, the posterolateral epiphyseal fragment and the posterior metaphyseal (Thurston-Holland) fragment remain united as a single fragment. In the three-part lateral variant, as observed in the present case, the anterolateral epiphyseal (Tillaux) fragment is fully detached, increasing fracture complexity and the technical demands of achieving anatomical reduction. This three-fragment configuration represents a minority pattern within an already uncommon injury [2,3].

Surgical management of displaced triplane fractures is classically performed through an anterior ankle incision, developed between the extensor hallucis longus and extensor digitorum longus, providing direct access to the sagittal and axial fracture components [1,4]. The posterior Thurston-Holland fragment, however, is conventionally reduced indirectly: the ankle is placed in dorsiflexion and internal rotation, and the fragment is reduced using the already-reduced anterior fragment as a positional reference, before being secured with a posteroanterior screw, without direct visualization [4].

A combined anterolateral and posterolateral approach has been described for an isolated pediatric triplane fracture requiring direct visualization of both the epiphyseal and metaphyseal components [5], and arthroscopically assisted techniques have more recently been reported to improve fracture visualization while minimizing surgical trauma in the pediatric transitional ankle fracture population [6]. In adult trimalleolar ankle fracture surgery, by contrast, the isolated posterolateral approach is increasingly favored specifically for small or comminuted posterior malleolar fragments, owing to superior direct visualization and more reliable anatomical reduction compared with indirect fixation [7]. Accordingly, fragment morphology on preoperative computed tomography, rather than fragment size alone, has been proposed as the more appropriate criterion to guide this decision [8].

We present the case of a pediatric patient who sustained a three-part triplane fracture of the distal tibia with a small, comminuted posterior Thurston-Holland fragment, managed entirely through a single posterolateral approach, and we discuss the comparative rationale for this technique relative to the anterior and combined approaches conventionally described for this fracture pattern.

## Case presentation

A 12-year-old male with no relevant medical history presented to the emergency department following a torsional fall injuring the left ankle. On physical examination, the patient presented with pain, edema, and visible deformity of the left ankle, with functional limitation preventing ambulation. The overlying skin was tense over the deformity, with associated blanching, raising concern for impending skin compromise but without frank open exposure. Neurovascular status of the distal extremity was intact.

Plain radiographs of the left ankle in anteroposterior and lateral projections identified a fracture line involving the distal tibial physis with extension into the metaphysis and epiphysis, consistent with a complex physeal fracture of triplane pattern, with displacement of the fragments and an articular surface step-off (Figures 1, 2).

**Figure 1:**
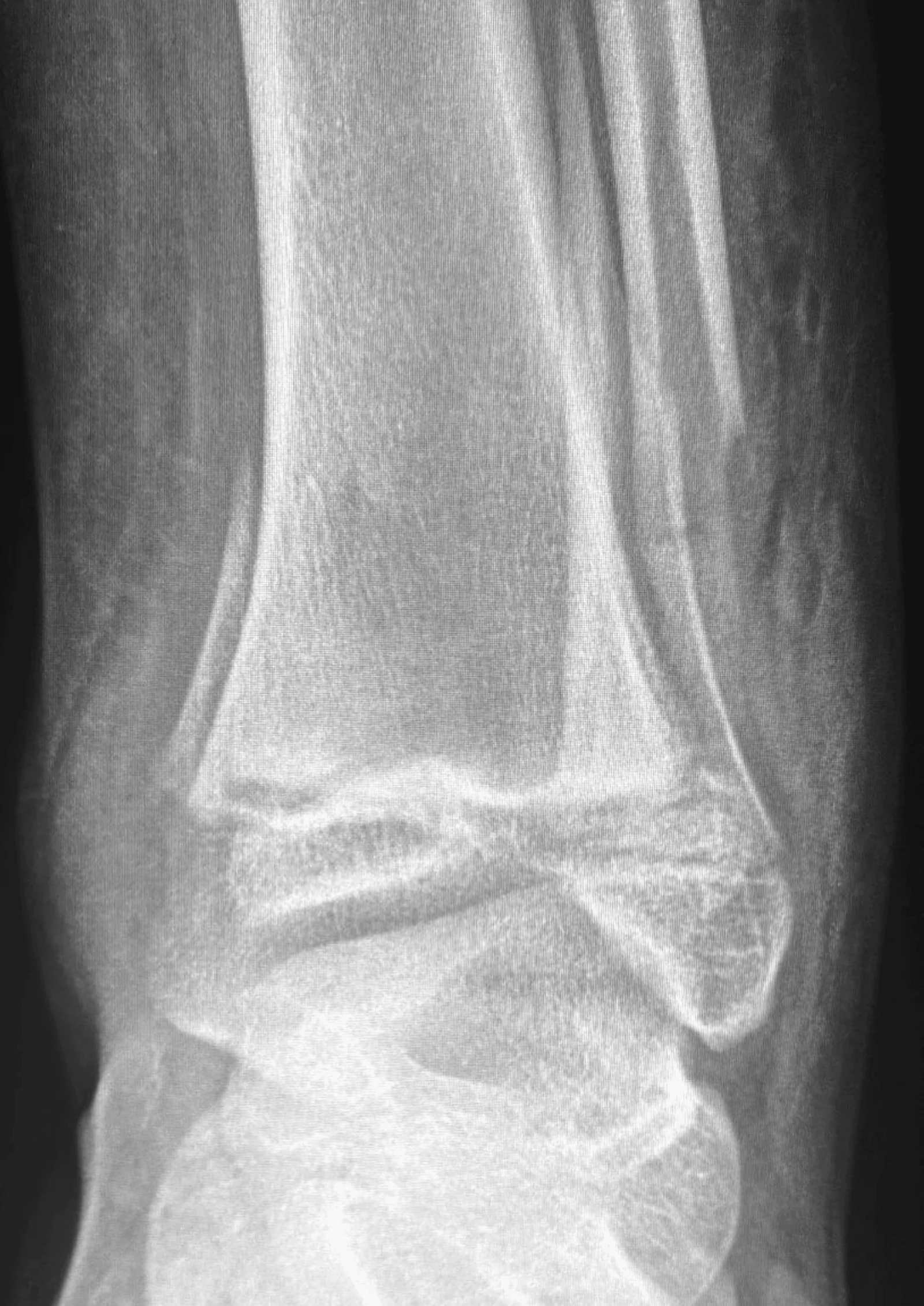
Anteroposterior radiograph of the left ankle demonstrating a fracture line involving the distal tibial physis, with extension into the metaphysis and epiphysis, configuring a complex physeal fracture of triplane pattern Fragment displacement with articular surface step-off is noted.

**Figure 2:**
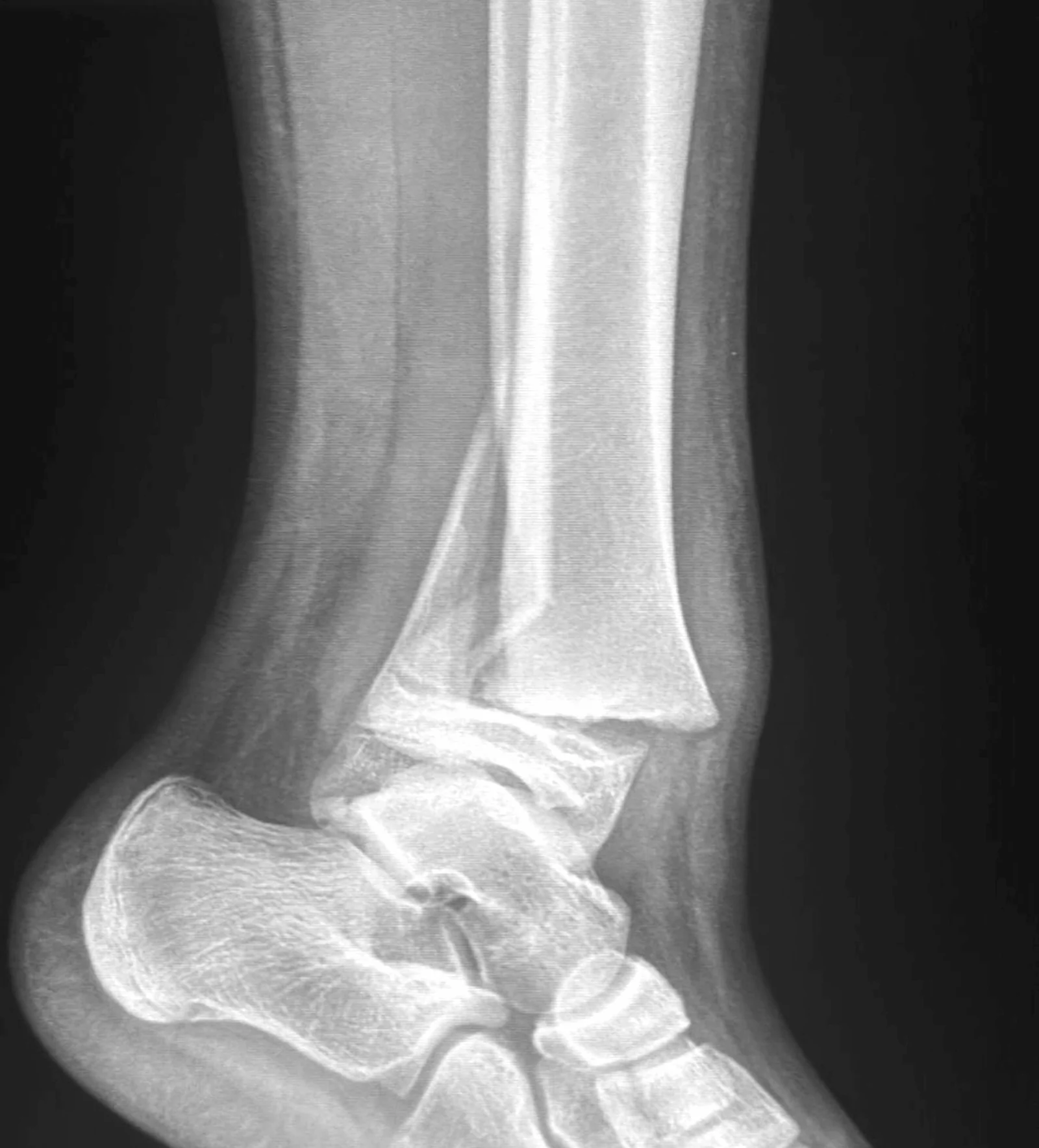
Lateral radiograph of the left ankle demonstrating a fracture line involving the distal tibial physis, with extension into the posterior metaphysis and involvement of the articular surface

Computed tomography was subsequently performed to further characterize the fracture, confirming a three-part triplane fracture of the distal tibia. Axial imaging demonstrated an oblique epiphyseal fracture line with separation of the anterolateral fragment, consistent with a Tillaux component, with approximately 3 mm of displacement and a 2-mm articular step-off, associated with a posterior metaphyseal component (Figure 3).

**Figure 3:**
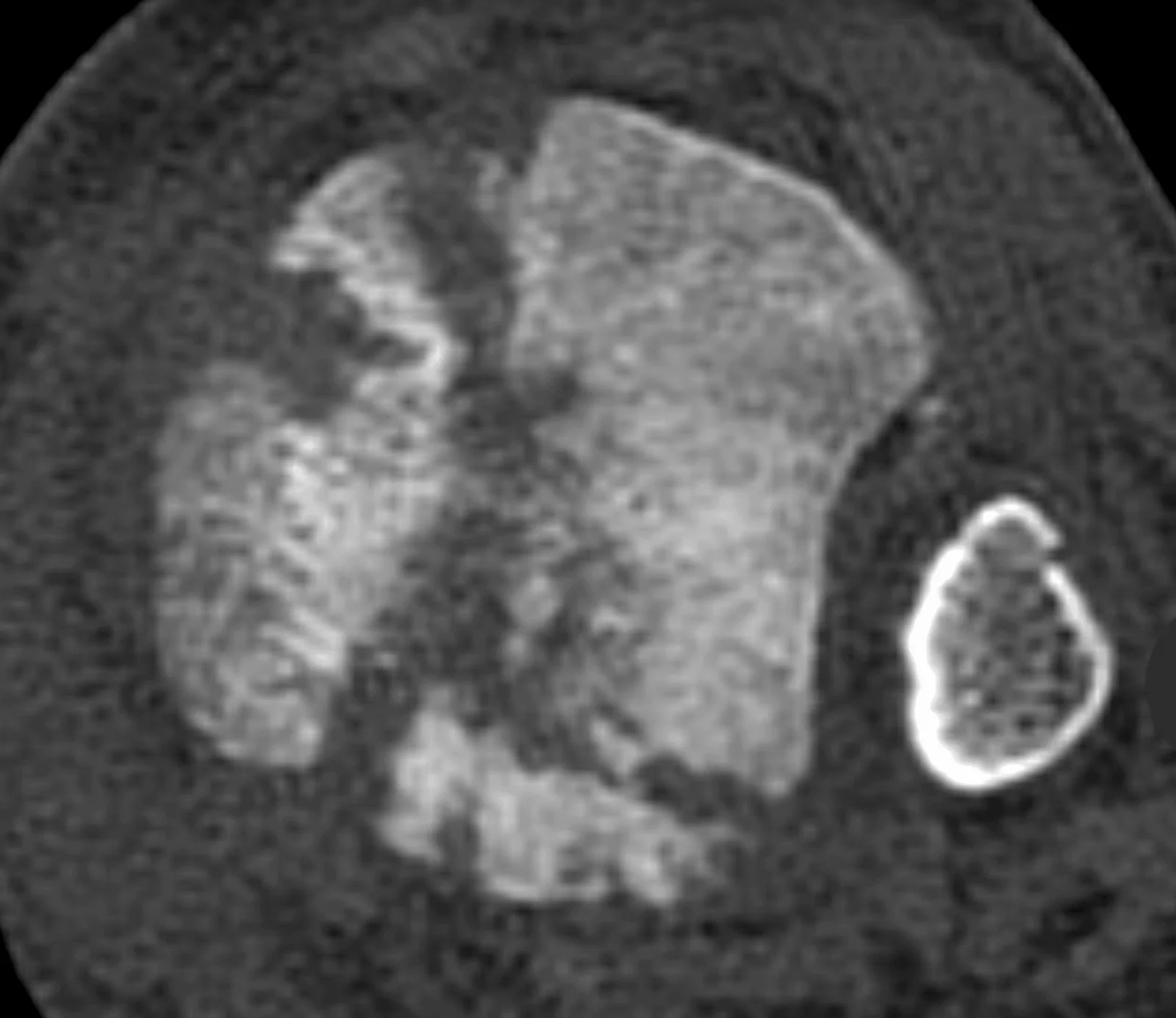
Computed tomography of the left ankle demonstrating a triplane fracture of the distal tibia with physeal involvement and intra-articular extension The axial plane identifies an oblique epiphyseal fracture line with separation of the anterolateral fragment, consistent with a Tillaux component, with approximately 3 mm of displacement and a 2-mm articular step-off, associated with a posterior metaphyseal component.

Given the degree of displacement and the soft-tissue compromise at risk, the patient underwent surgical fixation on the same day as the injury. The posterolateral approach additionally allowed direct fracture access away from the zone of greatest skin tension, avoiding additional incisions in an already compromised soft-tissue envelope. The patient was positioned prone, and open reduction and internal fixation was performed entirely through a single posterolateral approach, without an additional anterior incision. The internervous plane between the peroneus brevis and flexor hallucis longus was developed, with careful medial retraction of the flexor hallucis longus to protect the posterior tibial neurovascular bundle. Visualization of the anterolateral (Tillaux) fragment was achieved by extending the posterolateral incision anteriorly with subcutaneous soft-tissue mobilization, rather than through a separate anterior incision. All three fracture fragments were reduced simultaneously under continuous fluoroscopic guidance. The comminuted posterior Thurston-Holland fragment and the anterolateral (Tillaux) fragment were directly visualized through the posterolateral working window and stabilized with Kirschner wires. The medial epiphyseal fragment was fixed with a percutaneous, self-tapping headed screw. A posterior anatomical plate was applied without transgression of the physis, with epiphyseal self-tapping screws confined to the epiphysis and three Kirschner wires (two posterior, one medial), without evidence of articular penetration. Tibiotalar alignment and mortise congruence were restored (Figures 4, 5).

**Figure 4:**
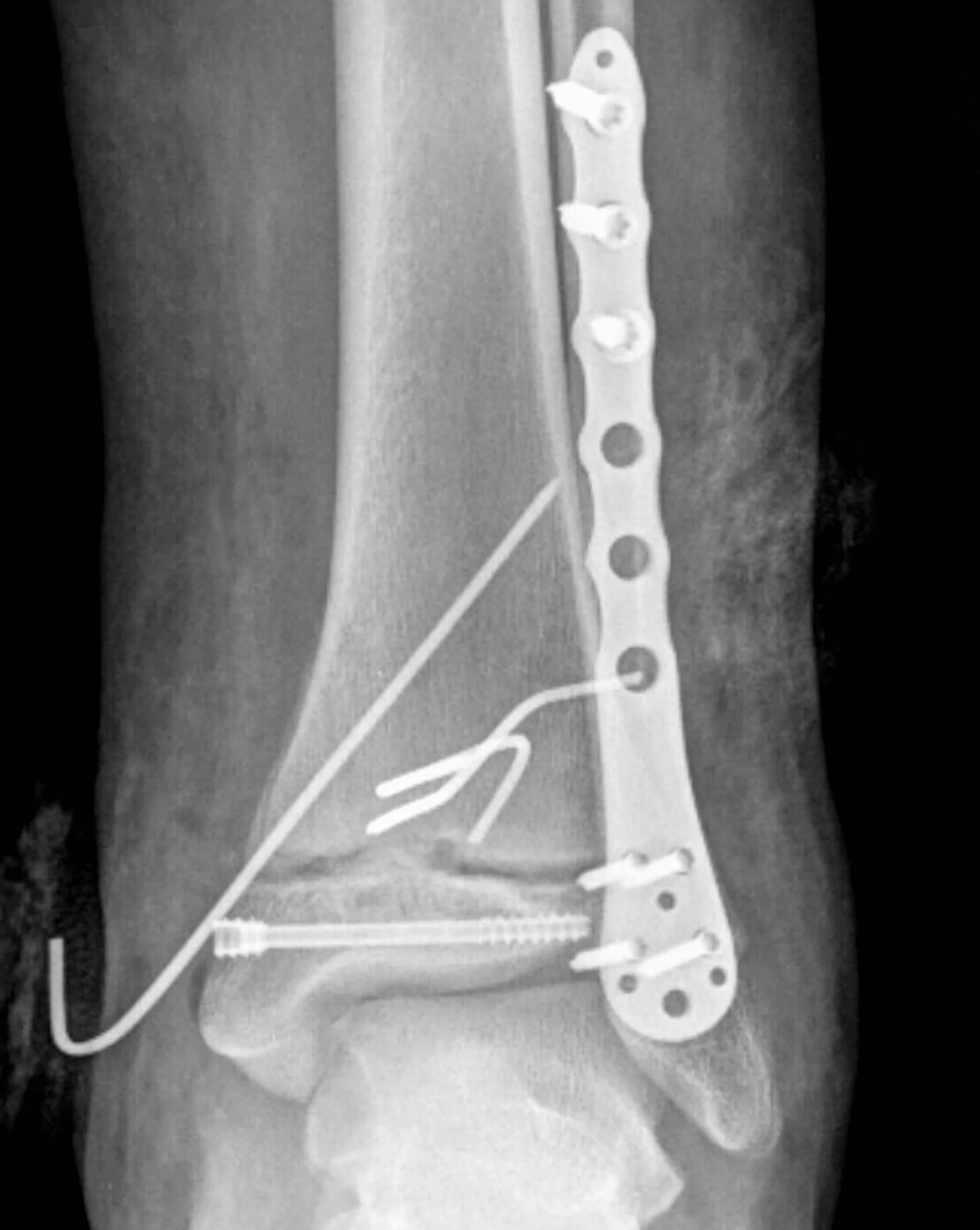
Anteroposterior radiograph following open reduction and internal fixation of the triplane fracture of the distal tibia The radiograph demonstrates adequate reduction and restoration of articular surface congruence. The posterior anatomical plate is in adequate position without transgression of the physis, associated with self-tapping screws confined to the epiphysis and three Kirschner wires (two posterior and one medial), without evidence of articular penetration. Tibiotalar alignment and mortise congruence are preserved.

**Figure 5:**
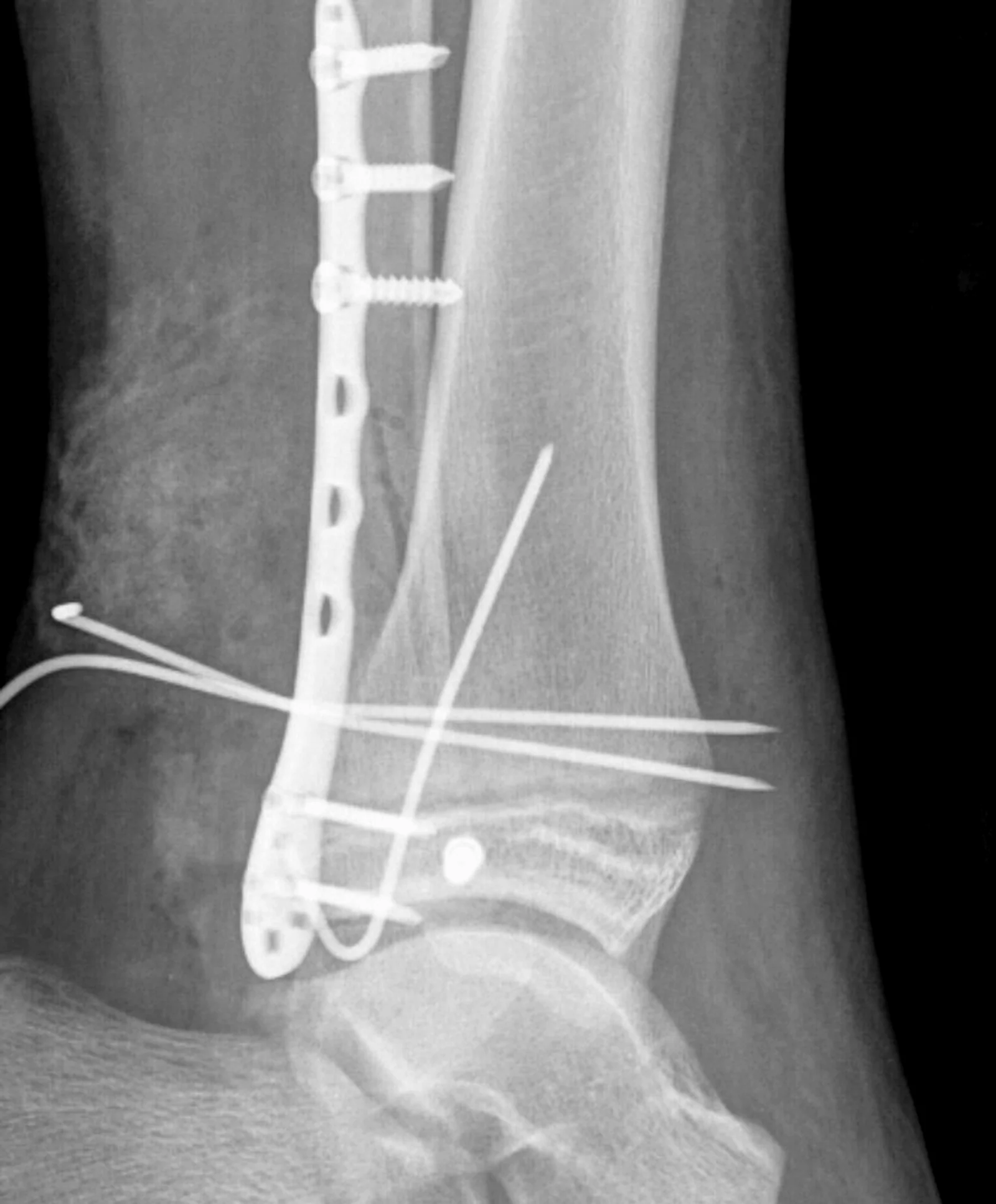
Lateral radiograph following open reduction and internal fixation, demonstrating adequate reduction of the sagittal component of the triplane fracture and restoration of articular congruence The posterior anatomical plate and fixation hardware are in adequate position, with epiphyseal screws without physeal or articular transgression and posterior Kirschner wires correctly positioned.

Postoperatively, the ankle was protected in a CAM (controlled ankle movement) walker boot. At six weeks postoperatively, the Kirschner wires were removed in the outpatient clinic following radiographic confirmation of partial fracture consolidation, at which point gradual partial weight-bearing was initiated with progressive loading in the orthopedic boot. Separately, at six months postoperatively, the epiphyseal self-tapping screw was removed following radiographic confirmation of complete fracture consolidation. Following screw removal, radiographs demonstrated a completely consolidated triplane fracture, with anatomical restoration of the epiphyseal-metaphyseal relationship, appropriate fragment alignment, and tibiotalar articular congruence. There was no residual displacement or articular step-off (0 mm), and the tibiofibular-talar mortise was preserved without axis alteration or signs of complication (Figure 6).

**Figure 6:**
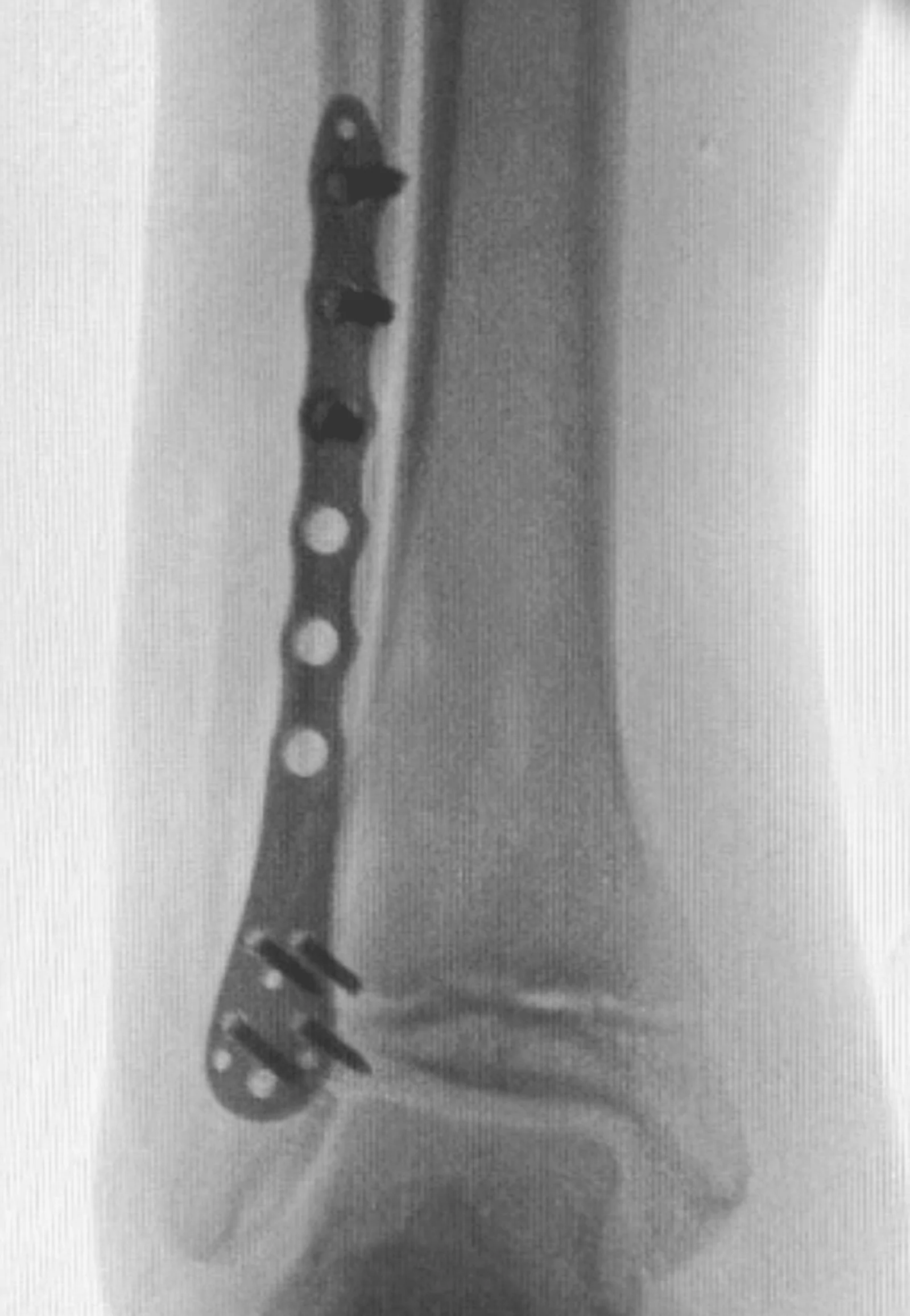
Anteroposterior radiograph at six months postoperatively, following removal of the self-tapping screw The triplane fracture of the distal tibia is completely consolidated, with anatomical restoration of the epiphyseal-metaphyseal relationship, adequate fragment alignment, and tibiotalar articular congruence, without residual displacement or articular step-off. The tibiofibular-talar mortise is preserved, without axis alteration or signs of complication.

At eight months postoperatively, the patient demonstrated 45° of plantar flexion and 20° of dorsiflexion, no pain, ambulation of four to six blocks without difficulty on any surface, no gait abnormality, normal hindfoot motion, and stable tibiotalar and subtalar joints on examination, with a well-aligned, plantigrade foot. The American Orthopaedic Foot and Ankle Society (AOFAS) Ankle-Hindfoot score [9] was 99 out of 100 points (40 points for pain, 10 for activity limitation, 4 for maximum walking distance, 5 for walking surfaces, 8 for gait abnormality, 8 for sagittal motion, 6 for hindfoot motion, 8 for hindfoot stability, and 10 for alignment), corresponding to an excellent functional outcome. Clinical and radiographic assessment at this visit showed no evidence of premature physeal closure or angular deformity.

## Discussion

Epidemiology

Triplane fractures represent approximately 5%-15% of pediatric intra-articular ankle injuries and occur almost exclusively in adolescents aged 12-15 years, with a slightly higher incidence in boys, coinciding precisely with the period of asymmetric distal tibial physeal closure [1,2]. The three-part lateral variant observed in the present case, in which the anterolateral Tillaux fragment is fully detached from the remaining epiphysis, represents a more complex pattern than the more common two-part configuration [2,3]. Although generally associated with favorable outcomes when appropriately managed, displaced triplane fractures can occasionally be complicated by neurovascular compromise, underscoring the importance of prompt recognition and careful surgical planning in this population [10]. In the present case, serial clinical and radiographic follow-up through eight months postoperatively showed no evidence of premature physeal closure or angular deformity, although longer-term surveillance would be needed to fully exclude late growth disturbance given the patient's skeletal immaturity.

Pathophysiology

The distal tibial physis closes in a predictable, asymmetric sequence, beginning centrally, proceeding medially, and concluding anterolaterally over approximately 18 months [1]. A torsional force applied during this transitional period propagates through the relatively weak, still-open portion of the physis in the axial plane, exiting through the stronger, already-closing metaphysis and epiphysis in the coronal and sagittal planes, respectively [1,4]. The comminution of the posterior Thurston-Holland fragment observed in the present case likely reflects the magnitude of the causative torsional force and the specific stage of physeal closure at the time of injury.

Diagnosis

Plain radiographs in multiple projections are typically sufficient to raise suspicion of a triplane fracture, but computed tomography is considered essential for adequately characterizing fragment number, displacement, and comminution, and is critical for surgical planning [1,4]. Plain radiographs reliably estimate only the overall size of the posterior fragment, while comminution and impaction are consistently underestimated and require CT for accurate assessment [11]. In the present case, computed tomography was essential not only for confirming the three-part pattern but specifically for identifying the comminution of the posterior fragment, a finding that directly informed the choice of a posterolateral surgical approach over the conventional anterior technique.

Differential diagnosis

The differential diagnosis of pediatric ankle injuries in this age group includes isolated Tillaux fractures, simple Salter-Harris III or IV fractures, and adult-pattern ankle fractures in patients with near-complete physeal closure. An isolated Tillaux fracture involves only the anterolateral epiphyseal fragment without a posterior metaphyseal component, while simple Salter-Harris fractures lack the multiplanar fracture propagation characteristic of triplane injuries. In the present case, computed tomography confirmed fracture lines through all three planes together with a distinct comminuted posterior fragment, confirming the diagnosis of a three-part triplane fracture.

Treatment

Displaced triplane fractures, defined as those with more than 2 mm of articular displacement, are generally managed operatively, with open reduction and internal fixation remaining the preferred strategy overall and surgical complications remaining minimal across the pediatric triplane literature. Articular displacement exceeding 2 mm is considered a critical threshold, as failure to achieve anatomical reduction beyond this point carries a recognized risk of permanent articular cartilage damage and subsequent post-traumatic osteoarthritis [1,2].

Each of the surgical approaches described for this fracture pattern carries distinct advantages and limitations, which merit individual consideration. The conventional anterior approach, developed between the extensor hallucis longus and extensor digitorum longus, offers direct visualization of the sagittal and axial fracture components and remains the most extensively validated technique for triplane fractures, with screw fixation indicated once articular displacement exceeds 2 mm [1,4]. However, this approach relies on indirect, "blind" reduction of the posterior Thurston-Holland fragment: the ankle is dorsiflexed and internally rotated, and the fragment is reduced using the already-reduced anterior fragment as a positional reference before a posteroanterior screw is placed, without the surgeon ever directly visualizing the posterior fracture surface [4]. This strategy performs reliably only when the posterior fragment is a single, congruent piece, since intraoperative fluoroscopy - much like plain radiographs preoperatively - is known to underestimate comminution and impaction of the posterior fragment compared with direct visualization or CT [11].

The combined anterolateral-posterolateral approach addresses this limitation directly by exposing both the epiphyseal and metaphyseal fragments through separate incisions, allowing true anatomical reduction of each component under direct vision, but at the cost of two distinct surgical windows and correspondingly greater soft-tissue dissection and operative time [5]. Arthroscopically assisted techniques represent a further refinement, minimizing surgical trauma while still allowing precise visualization of the articular reduction, with favorable functional outcomes reported at short-term follow-up [6]. However, arthroscopic techniques require specialized equipment and expertise and are best suited to fractures without significant metaphyseal comminution that would otherwise obstruct arthroscopic visualization or complicate percutaneous fixation.

The isolated posterolateral approach, well established in adult trimalleolar ankle fracture surgery, offers direct visualization specifically of the posterior fragment through the internervous plane between the peroneus brevis and flexor hallucis longus, and has been associated with a greater proportion of favorable range-of-motion outcomes and fewer complications, including osteoarthritis, compared with the posteromedial approach, although this early functional advantage does not appear to persist as a significant difference over longer-term follow-up [7]. Its principal limitation in the context of triplane fractures is anatomical rather than technical: fragments lying outside the posterior working window, such as the Tillaux fragment, are not routinely visualized unless the surgical window is deliberately extended anteriorly within the same approach, as was performed in the present case.

Underlying the choice between these approaches, a morphology-guided strategy - selecting the surgical approach based on posterior fragment size and comminution on preoperative computed tomography rather than on fracture classification or displacement alone - has been shown to better predict postoperative joint congruence and degenerative outcomes than fragment size in isolation [8]. This is consistent with findings from posterior malleolar fracture series, in which comminuted (intercalary) fragments are both more frequent in younger patients and significantly associated with the surgeon's choice of a posterolateral, rather than posteromedial or indirect, approach [12] - a pattern that closely parallels the clinical reasoning applied in the present pediatric case.

In the present case, the posterior Thurston-Holland fragment was small and comminuted, a scenario in which indirect reduction cannot be assumed to reliably restore articular congruence. Applying the morphology-guided rationale described above [8,12], a posterolateral approach was selected to allow direct visualization of the comminuted fragment. In this case, the same posterolateral working window also permitted direct visualization and fixation of the anterolateral Tillaux fragment, avoiding the additional anterior incision required in the combined technique described previously [5]. Fixation was achieved using Kirschner wires and a percutaneous screw, consistent with reported comparable outcomes between these two fixation methods [13].

We propose fragment morphology on preoperative computed tomography as a potentially useful, hypothesis-generating consideration, rather than an established recommendation, when selecting a surgical approach for pediatric triplane fractures. Rather than representing a limitation alone, this single case should be regarded as a foundational observation that prompts larger comparative series to formally evaluate a morphology-driven, single-approach fixation strategy. The relatively short follow-up period in this skeletally immature patient similarly underscores the need for extended follow-up in future studies to validate long-term physeal and functional outcomes.

The hybrid fixation construct used in this case was selected to address the distinct biomechanical demands of each fracture fragment. The posterior anatomical plate provided stable metaphyseal-diaphyseal anchorage away from the physis, resisting the shear and torsional forces at the primary fracture line. Kirschner wires were used for the comminuted posterior and Tillaux fragments, as their smaller size and irregular morphology were better suited to low-profile fixation without requiring additional bone stock for screw purchase. The percutaneous self-tapping screw was reserved for the medial epiphyseal fragment, providing interfragmentary compression across a discrete fracture line while remaining confined to the epiphysis and avoiding physeal transgression. This combination allowed each fragment to be fixed according to its individual size, morphology, and proximity to the physis, rather than applying a single fixation method uniformly across all three fragments [7,8,12].

## Conclusions

Pediatric triplane fractures are conventionally managed through anterolateral and/or anteromedial approaches, with the posterior Thurston-Holland fragment reduced indirectly using the anterior fragment as a reference. The present case illustrates that when this fragment is small and comminuted, indirect reduction may not reliably restore articular congruence, and a posterolateral approach may instead allow direct visualization and more reliable anatomical fixation. As with any single case report, these findings should be interpreted as hypothesis-generating rather than definitive; longer-term follow-up in a larger comparative series would further strengthen the evidence supporting this technique.

Furthermore, this case suggests that an isolated posterolateral approach may, in select three-part triplane fracture patterns, permit direct fixation of both the posterior and anterolateral fragments through a single surgical window, potentially reducing the soft-tissue dissection associated with combined anterior-posterior techniques described previously. Rather than representing a limitation alone, this single case should be regarded as a foundational observation prompting larger comparative series to formally evaluate a morphology-driven, single-approach fixation strategy. Fragment morphology on preoperative computed tomography, rather than fracture classification alone, should be considered when selecting a surgical approach in pediatric triplane fractures.
